# MRI-enhanced online adaptive radiotherapy by virtual integration of a standalone MRI simulator and CBCT-based treatment delivery

**DOI:** 10.1186/s13014-026-02887-w

**Published:** 2026-07-06

**Authors:** Silvia Fabiano, Klara Kefer, Michael Baumgartl, Hubert Gabrys, Riccardo Dal Bello, Elizabeth Denney, Michael Mayinger, Jens von der Grün, Lotte Wilke, Matthias Guckenberger, Stephanie Tanadini-Lang, Sebastian M. Christ

**Affiliations:** 1 Department of Radiation Oncology, University Hospital Zurich, Rämistrasse 100, Zurich, 8091 Switzerland; 2 Department of Radiation Oncology, Spital Thurgau, Frauenfeld, Switzerland; 3 Strahlentherapie, MVZ Gesundheit Nordhessen, Klinikum Kassel, Kassel, Germany

## Abstract

**Background and purpose:**

MR-guided radiotherapy (MRgRT) offers superior soft-tissue contrast and enables adaptive treatments which improve tumor targeting and organ-at-risk sparing. However, integrated MR-Linac systems remain financially and logistically inaccessible for many hospitals. To address this gap, we implemented a modular MRgRT workflow — termed the “Virtual MR-Linac”— which decouples MR imaging and treatment delivery, enabling daily adaptive radiotherapy (ART) using diagnostic-quality MRI.

**Materials and methods:**

Between November 2023 and January 2025, 31 male patients with pelvic lymph node metastases were treated using this workflow. Daily MR scans were acquired in treatment position using a 1.5T MR scanner. Contours were adapted on 2D T2 turbo-spin-echo images, while synthetic CTs generated from T1 DIXON sequences were used for dose calculation. Adapted plans were delivered on a C-arm Linac using CBCT imaging for positioning. Quality assurance included independent Monte Carlo dose recalculations, automated plan parameters verification and a plan complexity check using in-house scripting.

**Results:**

Across 99 adapted fractions, plan adaptation improved dosimetric outcomes, with a median 14% increase in Planning Target Volume (PTV) coverage and a 7% reduction in bowel D1cc (dose to 1 cc). Median times for MR imaging, contouring, and planning were 12, 12, and 20 min, respectively.

**Conclusions:**

The Virtual MR-Linac approach enables high-quality ART to compensate for inter-fractional motion without the costs and complexity of integrated systems. It improves resource utilization, maintains patient comfort, and offers a flexible, scalable solution for clinical implementation.

**Supplementary Information:**

The online version contains supplementary material available at https://doi.org/10.1186/s13014-026-02887-w.

## Introduction

Magnetic Resonance (MR) has become a benchmark in radiation oncology treatment planning, as it provides high spatiotemporal resolution, superior soft-tissue contrast compared to computed tomography (CT), and functional as well as biological tissue characterization capabilities [1]. Building on these advantages, MR-guided radiotherapy (MRgRT) has emerged as a promising approach for adaptive radiotherapy. In regions affected by substantial inter-fractional anatomical variability, daily changes in patient anatomy can considerably alter target position relative to organs at risk (OARs). Compared with CT-based imaging, the superior soft-tissue contrast of MRI enables more accurate target and OARs delineation, thereby facilitating daily anatomy-driven treatment adaption with greater confidence. This may enable more precise tumor targeting, reduced safety margins, and minimized damage to surrounding healthy tissues [2–6]. In addition, real-time imaging during treatment delivery further enables intra-fractional motion monitoring [7–9], offering the potential to improve patient outcomes and reduce side effects [10, 11].

However, the implementation of MRgRT faces significant financial and logistical challenges that limit its widespread adoption [12, 13]. The high manufacturing costs of MR-guided linear accelerator (MR-Linac) systems, driven by the complexity of integrating linear accelerator technology with MR functionality, pose obstacles to their broader commercial viability. Construction, purchase, and maintenance costs for these systems are approximately two to three times higher than those of standard CT-based linear accelerators, posing a substantial financial barrier for many departments [14]. Moreover, online adaptive radiotherapy (oART) with MR-Linacs is both resource- and time-intensive [15], further limiting patient throughput and overall accessibility.

Recently, CT-guided oART systems, including the *Varian Ethos* and *Elekta Evo* radiotherapy platforms, have been introduced. These systems integrate semi-automated anatomy detection and plan generation, enabling streamlined and fast adaptive workflows [16–20]. However, CT imaging is inherently limited by its lower soft-tissue contrast compared with MRI, which may reduce confidence in target and OARs delineation during daily adaptation, particularly in anatomically complex regions affected by substantial inter-fractional variability. To address this limitation, previous studies proposed a workflow combining weekly MR imaging with Cone Beam CT (CBCT)-based ART through a shuttle-based approach, thereby incorporating offline MR guidance into the adaptive process [21–23]. However, because MR imaging was performed only intermittently, this approach could not fully support daily anatomy-driven adaptation.

Building upon this approach, our team has developed an innovative MRgRT system design, termed “Virtual MR-Linac”, as part of the ARTEMIS Program (Adaptive Radiation Therapy Enhanced by Magnetic Resonance Imaging Systems). The system features a dedicated MR scanner in close proximity to multiple C-arm linear accelerators. This multi-room configuration enables an adaptive radiotherapy workflow, in which patients undergo daily MR-imaging in treatment position using a diagnostic MR scanner specifically tailored to radiotherapy needs, allowing daily anatomy-driven re-contouring and plan adaption based on diagnostic-quality MR-images. Adapted plans are generated on synthetic CT (sCT) images derived from the daily MRI and subsequently delivered on a C-arm linear accelerator using in-room CBCT-based image guidance.

We hypothesize that this approach preserves the clinical advantages of MRgRT by enabling daily adaptive treatments based on high-quality MR-imaging, while providing a more flexible and cost-effective alternative to fully integrated MR-Linac systems. Compared with MR-Linac technology, the proposed approach has the potential to streamline resource utilization, increase patient throughput, and leverage the superior imaging performance of a diagnostic MR as an integral component of the ART workflow.

To support the clinical implementation of this workflow, our research group has recently conducted a Failure Modes and Effects Analysis (FMEA), with the goal of ensuring patient safety and operational efficiency [24].

In this study, we report the initial clinical experience with the treatment of 31 male patients with pelvic lymph node lesions using the Virtual MR-Linac approach. We evaluate its dosimetric benefits and the adaption time.

## Materials and methods

### Patient cohort and prescriptions

This retrospective single-center analysis was approved by the Swiss Ethics Committee (BASEC: 2018 − 01794) prior to study initiation. All patients provided informed consent to undergo MR-guided treatment within the ARTEMIS program and to participate in the study.

The ART workflow using a virtual MR-linac was applied to 31 male patients with pelvic lymph node metastases who were eligible for MR-linac treatment. The median PTV volume was 4.51 cc (IQR: 3.16–13.23 cc).

The majority of patients (52%) were prescribed 10 Gy in 2 fractions, while 35% (11 patients) received 35 Gy in 5 fractions. The remaining patients were prescribed a single fraction of 5 Gy (2 patients; 6%), 4 fractions of 5 Gy (1 patient; 3%), or 6 fractions of 6 Gy (1 patient; 3%). An 80% prescription isodose line was consistently used.

OAR constraints primarily focused on the bowel (including sigmoid), with limits of D1cc < 30 Gy and D0.1cc < 33 Gy for fractionation schemes of 4–6 fractions. For treatments delivered in 1–2 fractions, avoidance of bowel hotspots was ensured.

### ART workflow using the virtual MR-Linac

#### Base plan preparation

For treatment planning, patients undergo 2D T2 turbo-spin-echo and 3D T1-DIXON MRI scans in treatment position using the MAGNETOM SOLA 1.5T MR scanner (Siemens Healthineers AG, Erlangen, Germany), located in our radiation oncology department. Detailed sequence parameters are provided in Supplementary Table T1. A sCT is generated at the scanner console using the T1-DIXON data. The sCT is then sent to MIM Software^®^ (v. 7.4.2, MIM Software Inc., Cleveland, OH, USA) for resampling to a 1 mm resolution and for auto-contouring of the main organs at risk (OARs), including the rectum, bladder, and bowel. MIM also automatically transfers the MR and sCT images into ARIA, with all images sharing the same frame of reference, eliminating the need for additional co-registrations. An experienced radiation oncologist reviews the quality of the intrinsic registrations and subsequently contours the target volumes on the sCT, using the T2-weighted image for guidance.

Next, a dosimetrist creates a stereotactic body radiotherapy (SBRT) plan using the Eclipse (v. 16.1, Varian Medical Systems, Palo Alto, CA, USA) treatment planning system, following a checklist to ensure compliance with internal quality standards [24]. Treatment plans are generated using institutional templates that define beam configuration (number of beams, directions, and energies) as well as optimization objectives and clinical goals. Dose calculation is performed on the sCT using the AcurosXB, v.16.1.0, algorithm (Varian Medical Systems). Plans are normalized to the Planning Target Volume (PTV), ensuring that 100% of the prescribed dose covers at least 98% of the PTV, with a maximum dose of 127% of the prescribed dose within the Gross Tumor Volume (GTV) (corresponding to a prescription isodose line of 80%).

#### Treatment adaption and delivery

**Fig. 1:**
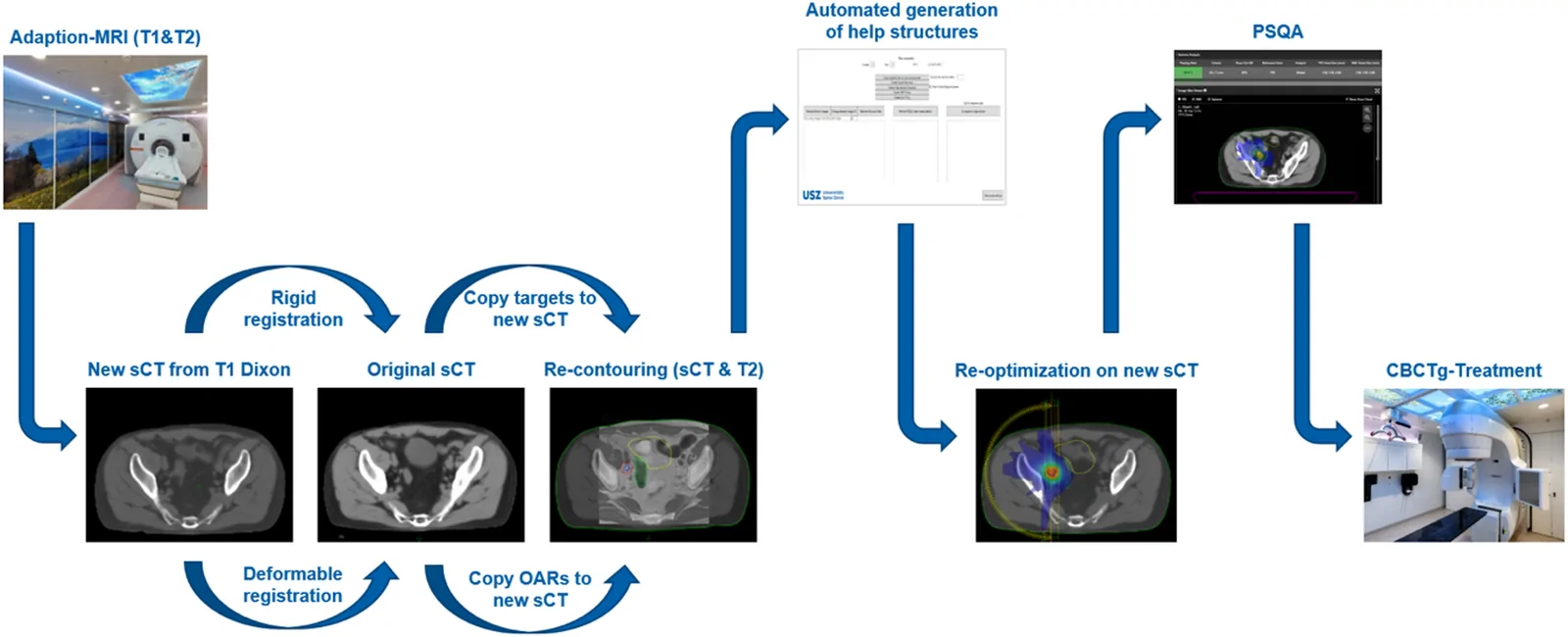
Cone Beam Computed Tomography (CBCT)-guided adaptive radiotherapy with a daily magnetic resonance (MR)-informed workflow [24]

As outlined in Fig. 1, the adaptive workflow includes the following steps and was applied to all 31 patients and 99 fractions. First, T2-weighted and T1-DIXON MRI sequences are acquired in treatment position using the 1.5T MAGNETOM SOLA MR scanner. A smaller field of view (FOV) in the superior-inferior direction minimizes imaging time to under 15 min. At the scanner, the system automatically generates a new sCT based on the daily anatomy. Afterwards, all images are manually sent to ARIA and automatically renamed according to institutional guidelines using an in-house developed Eclipse Scripting API (ESAPI) script. This script also resamples the sCT to a 1 mm resolution. Although all images share the same frame of reference, minor patient motion between MRI sequences may occur during acquisition. Therefore, the quality of the intrinsic registration is systematically verified by an experienced radiation oncologist. If clinically relevant misalignment between MRI sequences is observed, the image frame of reference can be reassigned and manual registration is performed prior to contour adaptation.

Next, the daily sCT is registered to the original planning sCT. The original target contours are transferred onto the new sCT using rigid registration, whereas the OAR contours are transferred using deformable registration. A physician then adapts the GTV on the sCT using the T2-weighted image for guidance and generates the PTV. Additionally, any OARs within a 2 cm margin around the GTV, are also adapted as needed.

A physicist then uses the ESAPI script to automatically copy the original treatment plan onto the new sCT, attach the adapted structure set, and create auxiliary structures necessary for plan optimization. The physicist can either recalculate the base plan on the daily anatomy or perform a full optimization, informed by changes in target–OAR geometry and the extent of contour modifications. When re-optimization is required, the original optimization objectives are used as a starting point and may be manually adjusted until an acceptable plan that meets clinical goals is achieved. Finally, the physician approves the selected plan following a dosimetric assessment.

To ensure accurate and safe treatment delivery, multiple verification checks are performed prior to beam-on, as described in section “Plan-specific quality assurance”.

Patients are ambulatory between imaging and treatment delivery and are repositioned on the treatment couch prior to irradiation. After completing the adaptive procedure and before delivering radiation, a verification CBCT scan is acquired to aid patient positioning at a TrueBeam Linac equipped with either a High Definition Multileaf Collimator (HD-MLC) or a Millennium MLC (Varian Medical Systems, Palo Alto, CA, USA).

#### Plan-specific quality assurance

Plan-specific quality assurance (QA) for both the base and adapted plans involves the following steps:

- Independent Monte Carlo dose calculation: Performed using the SciMoCa software (Radialogica LLC, vs. 1.8.1.5721). Quality assurance (QA) is considered successful if the gamma index analysis between TPS and SciMoCa doses achieves a pass rate greater than 95%, based on a 3%/1 mm criterion (for global normalization with a 20% dose cut-off);
- MLC opening complexity test: Conducted via the ESAPI script to identify potentially complex plans, defined by a mean MLC opening per beam (MMO) of less than 8 mm [25]. Plans meeting this criterion require additional portal dosimetry measurements; and.
- Plan recalculation with a water override of the body: This step serves as a QA measure for the sCT [26]. A criterion of [-1%; +4%] for the PTV mean dose difference is applied to determine a successful QA.
- Automated verification of multiple parameters (e.g., photon energy, dose rate, calculation grid size and model, treatment machine).

Additionally, the ESAPI script automatically verifies plan normalization and conducts a comparative analysis of key parameters, including dose per fraction, target volumes (with an internal threshold of 20%), and monitor units (MUs) (with an internal threshold of 20%), against the base plan.

### Data analysis

A dosimetric evaluation was performed for the 31 included patients with pelvic lymph node metastases. The dose distributions of the base plan, the adapted plan, and the base plan recalculated on the daily sCTs were compared to assess the impact of adaptation on target coverage and OAR sparing. This analysis was complemented by a time evaluation of the adaptive workflow, in which five phases were measured:

I. Imaging time: the interval from the start of MRI acquisition to the completion of sCT generation;
II. Image importing and registration time: the time required to import, prepare, and register the images in ARIA;
III. Contouring time: the time required to adapt the contours;
IV. Planning time: the time taken to adapt the treatment plan until final approval; and.
V. Documentation time: the time needed for plan-specific QA and documentation following plan approval before treatment delivery.

Additionally, the total session time was defined as the interval from the start of MRI acquisition to the completion of the radiation treatment, and notably included any delays such as waiting times, or workflow obstacles.

Portal dosimetry measurements, when performed, were acquired before treatment delivery and were included in phase V.

Finally, a comparative evaluation of resource utilization was conducted using a 0.35T MR-Linac workflow as benchmark.

## Results

Our department began treating patients using this workflow in November 2023. By January 2025, 31 male patients with pelvic lymph node metastases had completed treatment. In total, 99 fractions were adapted for the 31 patients, and treatment was completed as planned in all cases. Portal dosimetry measurements were performed in 2 out of 99 fractions (2%) and were acquired before treatment.

**Fig. 2:**
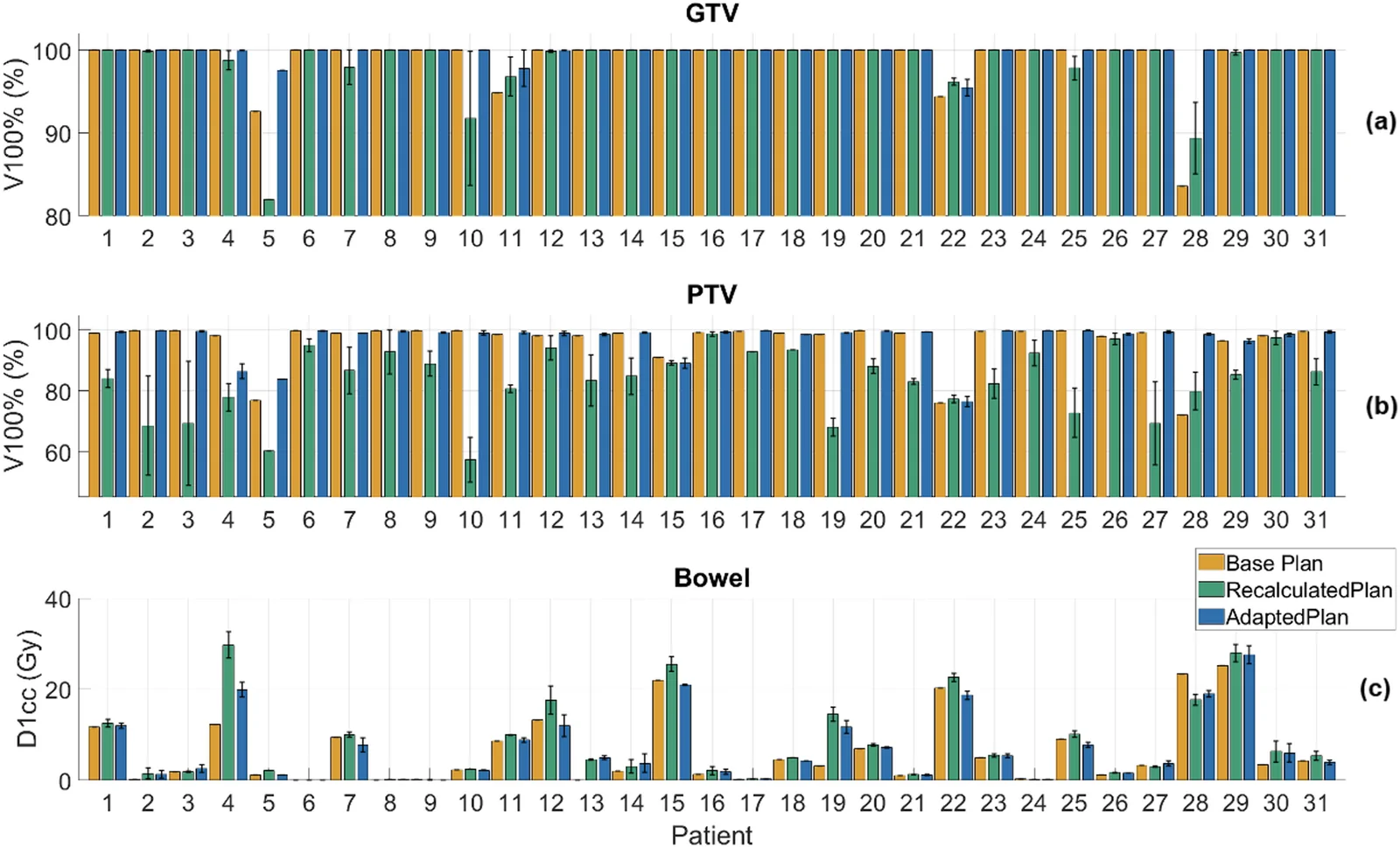
(**a**) Average Gross Tumor Volume (GTV) and Planning Target Volume (PTV) receiving the prescribed dose (**a**-**b**), as well as average dose to 1 cc of the bowel (**c**) across all patients for the base plan (orange), the base plan recalculated on the daily sCT (green), and the adapted plans (blue)

The dosimetric benefits of the adaptive approach are highlighted in Fig. 2 (with expanded subgroup analysis by fractionation scheme available in the Supplementary Figures F1 and F2). For each patient, Figs. 2a-c show the average GTV and PTV volumes receiving the prescribed dose, as well as the average dose to 1 cc of the bowel, for the base plan, the adapted plans, and the base plan recalculated on the daily anatomy. Plan adaptation resulted in improved bowel sparing in most cases (Fig. 2c). For patients in whom the bowel already received low doses, adaptation primarily enhanced target coverage (Fig. 2a and b). Specifically, plan adaptation led to a median increase in PTV coverage of 14% and a reduction in bowel dose of 7%.

**Fig. 3:**
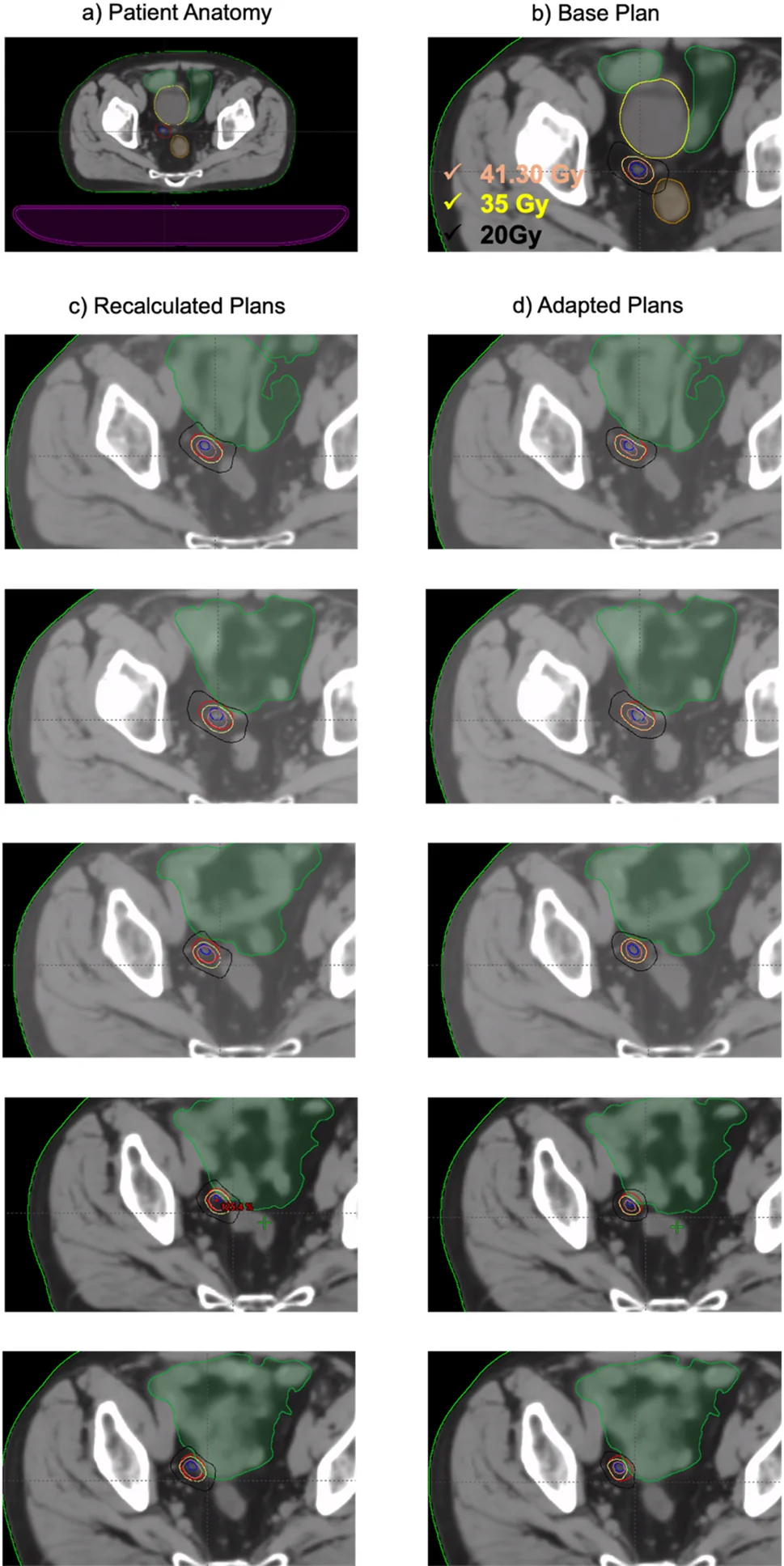
Representative patient case illustrating the impact of daily plan adaptation. (**a**) Patient anatomy in the baseline sCT with the Gross Tumor Volume (blue), Planning Target Volume (red), and bowel (green) delineated. (**b**) Base SBRT plan. Subsequent rows show daily sCT images for each of the five treatment fractions. For each fraction, the base plan recalculated on the daily images is shown in the left column (**c**), and the adapted plan is shown in the right column (**d**)

A representative case (Patient 4) is shown in Fig. 3, illustrating how the Virtual MR-Linac workflow can compensate for inter-fractional anatomical variations, maintaining adequate target coverage while improving bowel sparing. In the original plan, the bowel was located well away from the target volume (Fig. 3a and b). During all five treatment sessions, however, the bowel moved into close proximity to the target and partially overlapped with the PTV. When the original plan was recalculated on the daily sCTs, these anatomical changes resulted in both increased bowel irradiation and reduced dose conformality, leading to a decrease in PTV coverage from 98.2% in the original plan to 78% in the recalculated plans (Figs. 3c). By adapting the plan each day, mean PTV coverage was improved to 86% across all fractions. GTV coverage remained high, with the volume receiving the prescribed dose being 100% in the original plan, 99% in the recalculated plans, and 100% in the adapted plans. At the same time, plan adaptation reduced the average dose to 1 cc of the bowel from 30 Gy in the recalculated plans to 20 Gy in the adapted plans (Figs. 3d).

**Fig. 4:**
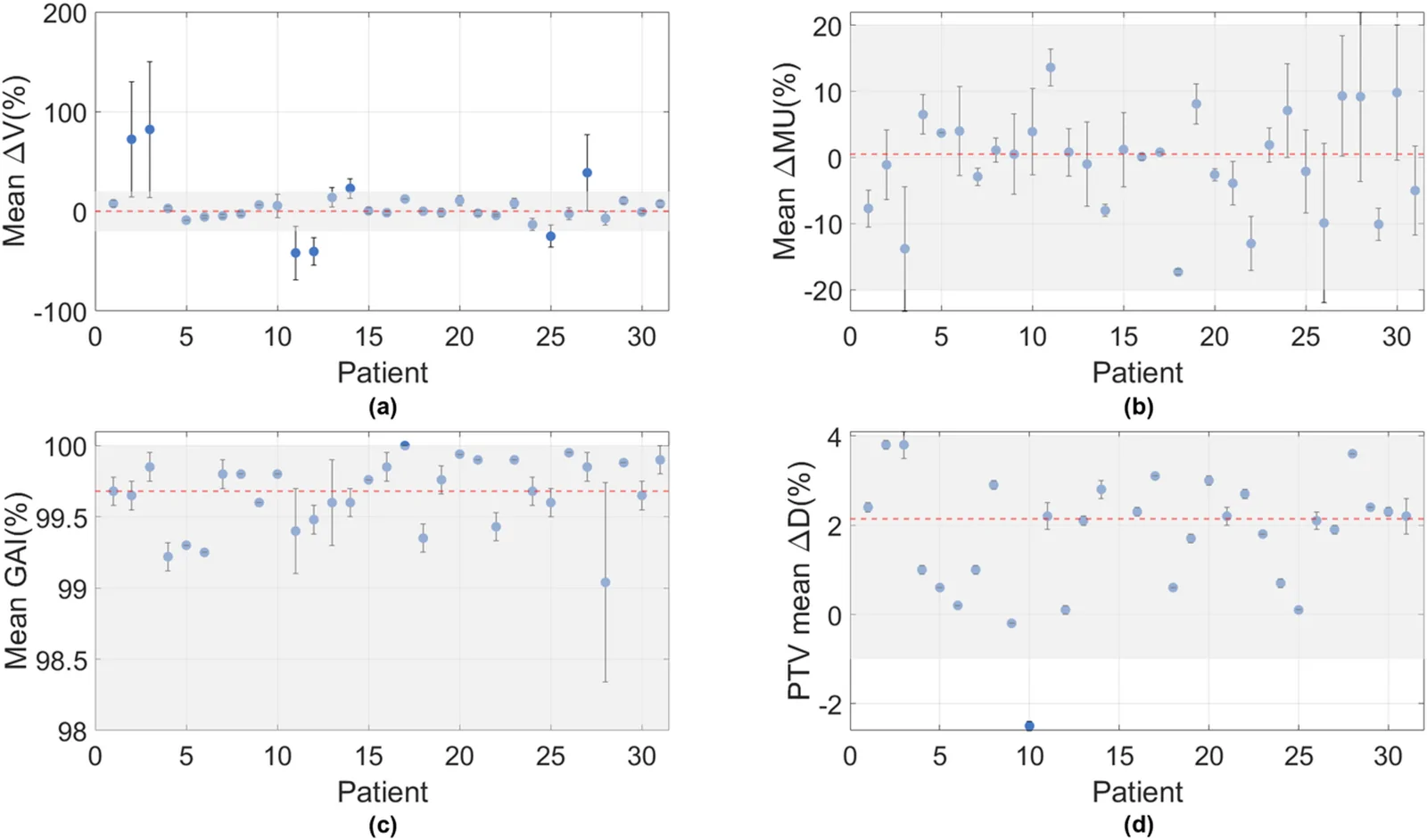
(**a**) Average deviation in planning target volume (PTV) volume, (**b**) average change in monitor units (MU), (**c**) average gamma agreement index (GAI) between treatment planning system and independent Monte Carlo dose recalculation, and (**d**) average deviation in PTV mean dose after plan recalculation with body water override for all patients. Results in (**a**) and (**b**) are reported relative to the base plan, while (**c**) and (**d**) are derived from the daily QA procedures. The grey shaded bands represent internal tolerances, and the red dashed lines indicate median values

The plan-specific QA results for all patients are shown in Fig. 4. Relative changes in PTV volume and MUs compared to the base plan were generally within 20% (Figs. 4a and b), with larger volume changes observed only in small tumors (< 8 cc). Independent Monte Carlo dose calculations yielded a median gamma passing rate of 99.7% (Fig. 4c). QA for the sCT was within the tolerance range of (-1%; 4%) for all patients except one (Fig. 4d). For this overweight patient, plan recalculation using a bulk density override confirmed the correctness of the sCT [26].

**Fig. 5:**
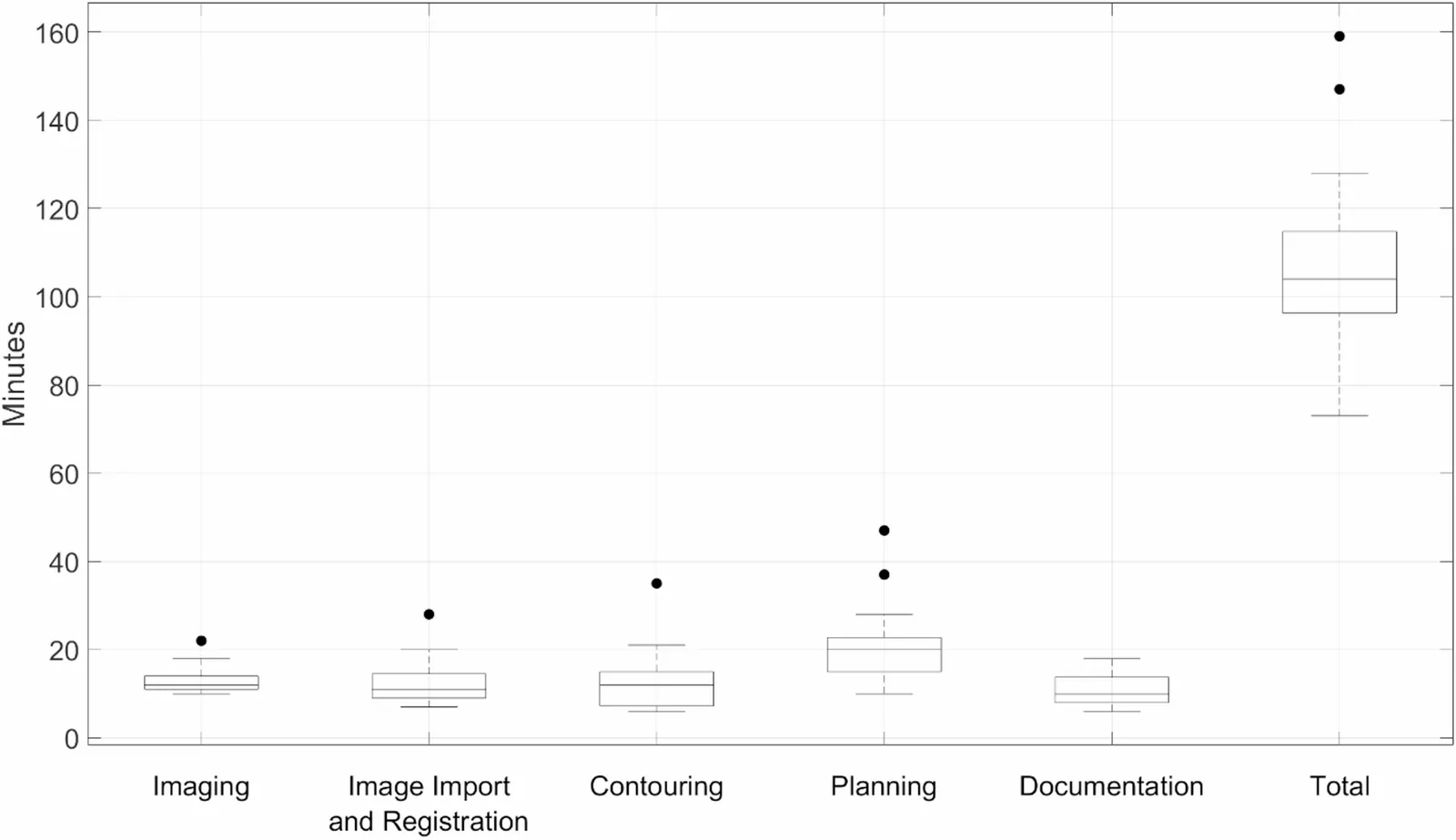
Median duration of the five phases of adaptive sessions. Time definitions are detailed in section “Data analysis”. “Total time” represents the interval from MRI acquisition to the completion of radiation treatment

The median total session time was 104 min (IQR: 96–114 min), with outliers resulting from delays or workflow interruptions, such as machine unavailability during concurrent treatment (Fig. 5). The median times for individual workflow phases were as follows: imaging, 12 min (IQR: 11–14); image preparation, 11 min (IQR: 9–14); contouring, 12 min (IQR: 8–15); planning, 20 min (IQR: 15–22); and documentation, 11 min (IQR: 8–13) (Fig. 5).

In order to benchmark resource utilization against the 0.35T MR-Linac workflow, 18 patients with pelvic lymph node metastases, comprising a total of 81 adapted fractions, were evaluated. This workflow proved to be more streamlined, with shorter times (Table 1). However, the Virtual MR-Linac approach—despite requiring longer adaptation times—is more resource-efficient in terms of equipment usage (Figure F3, Supplementary Material), with the MR scanner and the Linac being used for only approximately 12 and 15 min, respectively (Table 1).

**Table 1:** Comparison of the median of average values for image acquisition time, adaptation time (including contouring, plan re-optimization, and plan-specific quality assurance), and treatment delivery time between the Virtual MR-Linac and a 0.35T MR-Linac

	Adaptation session times (min)		
	Imaging Time	Adaptation Time	RT Time
Virtual MR-Linac	12 (11–14)	53 (39–64)	15 (13–18)
MR-Linac	9 (8–11)	16 (14–19)	16 (14–18)

Data are based on 31 patients (99 adapted fractions) treated with the Virtual MR-Linac workflow and 18 patients (81 adapted fractions) treated with the 0.35T MR-Linac workflow. Values in parentheses indicate interquartile ranges

## Discussion

Between November 2023 and January 2025, 31 male patients with pelvic lymph node metastases were successfully treated using a Virtual MR-Linac at our department. Most patients received either 10 Gy in 2 fractions or 35 Gy in 5 fractions (87%). Dose was prescribed to the 80% isodose line for all patients, and all fractions were adapted, with treatments completed as planned. While adaptation was performed for all fractions in this study, the modular design of the workflow would also allow adaptation decisions to be made at different stages of the process.

Dose re-optimization was required for all fractions, either to restore target coverage or to improve OAR sparing, due to the base plans’ lack of robustness in a population exhibiting considerable inter-fractional anatomical variability. Pelvic lymph node metastases are particularly susceptible to internal organ motion driven by day-to-day variations in bladder, bowel, and rectal filling. Patients were therefore instructed to attend treatment with an empty rectum and a comfortably full bladder, achieved by drinking approximately one glass of water 45 min prior to the session. No additional dietary restrictions or bowel preparation measures were implemented. In this setting, the use of diagnostic-quality daily MR imaging facilitated anatomy-driven contour adaptation with improved confidence in target and OAR delineation. Plan adaptation ultimately resulted in a median increase in target coverage of 14% and a median reduction in bowel dose of 7%, underscoring the potential clinical benefits of daily adaptation.

These findings are consistent with prior studies. For example, *Winkel et al.* [27] reported target coverage improvements of 10–15% with daily MR-guided adaptation for pelvic nodes, while *Tetar et al.* [28]. observed significant bowel dose reductions through adaptation, with potential implications for reducing gastrointestinal toxicity. Given the established association between suboptimal target dosing and inferior local control in nodal metastases, the observed increase in PTV coverage may translate to improved tumor control [29–31]. Similarly, minimizing bowel dose is critical, as late GI toxicity remains a dose-limiting factor in pelvic RT, particularly when treating nodal disease adjacent to mobile bowel loops. In this context, our results further support the growing consensus that daily adaptation is not merely dosimetrically superior, but also clinically meaningful in improving therapeutic ratios for patients receiving pelvic nodal radiotherapy.

The median adaptive session time was 104 min, which currently exceeds the session durations reported for oART with MR-Linacs across various indications and protocols [32–34]. We acknowledge that these session times reflect an early-phase implementation and that further workflow optimization is essential both for clinical scalability and for minimizing the potential impact of intrafraction anatomical variation [35]. Nevertheless, because MRI acquisition and treatment delivery are physically decoupled, the workflow limits the time patients spend immobilized on the treatment table to less than 30 min, similar to some optimized MR-Linac workflows [36]. However, in contrast to MR-Linacs, where continuous machine occupancy is required throughout the adaptive session, the modular design additionally enables parallel imaging and treatment, a unique feature of the Virtual MR-Linac.

In fact, our comparative evaluation of resource utilization revealed that, while the MR-Linac workflow offers a streamlined and integrated solution, the Virtual MR-Linac approach allows for more flexible use of clinical resources. By separating the imaging and treatment steps, this workflow has the potential to increase patient throughput—reserving the MR-Linac for cases requiring intra-fractional tumor monitoring, while treating patients who can tolerate longer adaptation times with the Virtual MR-Linac.

Because patients are ambulatory between imaging and treatment, we acknowledge that intrafraction anatomical changes may partially reduce the dosimetric benefit observed in the adapted plans. Therefore, the results presented in Fig. 2 should be interpreted as a best case estimate of the dosimetric benefit under the current workflow implementation. Assessment of intrafraction motion between planning MRI and pre-treatment CBCT imaging represents an important step for this technology. However, this was beyond the scope of the present feasibility study and is instead a prespecified endpoint of a future prospective clinical trial. Importantly, substantial day-to-day anatomical variability remains a major challenge in pelvic nodal radiotherapy and was the primary motivation for implementing daily adaptive replanning in this study. In addition, qualitative review of the verification CBCTs revealed no major anatomical changes that prevented treatment delivery using the adapted plans.

Several factors were identified as important contributors to the overall session duration. Although the minimal MRI acquisition protocol itself requires approximately 6 min and 19 s, the total imaging time additionally includes acquisition of supplementary slices when needed, and generation of the sCT used for planning. Currently, the sCT is generated from T1-weighted images, requiring verification of the T1–T2 registration before contouring. Generating the sCT directly from T2-weighted images could eliminate this step and further reduce imaging time. Additional delays resulted from coordinating MRI acquisition with treatment machine availability. Dynamic allocation of the C-arm Linac based on expected availability may further improve efficiency. At the Linac, image matching was performed between the sCT and CBCT, whereas direct T2–CBCT matching would be preferable. Nevertheless, lymph nodes were clearly visible on the CBCTs, increasing our confidence not only in contour adaptation and dose calculation, but also in patient positioning. An example of a sCT–CBCT match is provided in the Supplementary Material (Figure F4). Finally, an initial learning curve further contributed to delays as the team became familiar with the workflow.

The Supplementary Figure F5 summarizes the workflow steps that are currently being automated using Python- and ESAPI-based scripting tools to reduce manual workload, standardize planning procedures, improve workflow robustness, and shift staff effort from manual execution toward verification and approval tasks. Once fully optimized, we anticipate median total session times below 40 min.

One limitation of our workflow is the absence of real-time imaging during beam-on time, a feature available in the MR-Linac through cine-MRI for tumor tracking during radiotherapy, allowing ART to compensate for inter-fractional changes but not intra-fractional motion. Furthermore, the workflow relies on intrinsic registration between MRI sequences acquired over a period of up to 12 min, during which patient motion may occur. Although all images shared the same frame of reference, small inter-sequence shifts cannot be entirely excluded. To mitigate this, all registrations were systematically reviewed by an experienced radiation oncologist, with the option to perform manual re-registration if required. In this cohort, no clinically relevant misregistrations were observed that necessitated correction. In addition, neither a second MRI immediately prior to treatment nor post-treatment imaging for dose verification was acquired in this study; both approaches will be evaluated in future studies.

Despite these limitations, the modular design of our system offers distinct advantages: it could be more easily implemented in standard radiotherapy departments, as most already possess the necessary hardware, unlike MR-Linacs. The main requirement is a dedicated software, which, once available, could be rapidly deployed. In addition, the system provides greater flexibility through the independent operation of each unit and allows for high-quality MRI for plan adaptation. These features make such a modular system a promising approach for enhancing accessibility and optimizing resource utilization.

For ART to achieve broader clinical adoption, cost-effective solutions must demonstrate clear dosimetric and/or clinical benefits in prospective trials. At our institution, we have opened the MACULA trial, a phase 1 clinical study assessing the feasibility and efficacy of MRgRT for pelvic and para-aortic lymph node metastases using a Virtual MR-Linac, combined with an AirShuttle system for patient transfer between imaging and treatment rooms. The latter may even further enhance workflow efficiency in a future, more time-efficient implementation of the workflow by eliminating the need for patient repositioning.

In conclusion, this study demonstrates that MRgRT using a Virtual MR-Linac is clinically feasible for the treatment of pelvic lymph node lesions. The approach may offer dosimetric benefits through daily anatomy-driven adaption based on diagnostic-quality MR-imaging, including improved target coverage and enhanced bowel sparing. Compared to integrated MR-Linac systems, the modular design provides a flexible and potentially more accessible solution for radiotherapy departments, optimizing resource utilization and improving patient comfort by minimizing time on the treatment table. Moving forward, we plan to expand this workflow to include tumor sites commonly treated on the MR-linac system at our institution, such as thoracic or abdominal tumors, to further assess its clinical versatility and extend its application across a wider range of disease sites.

## Supplementary Information

Below is the link to the electronic supplementary material.

Supplementary Material 1

## Data Availability

All data used and/or analysed during the current study are available from the corresponding author on reasonable request.

## References

[1] Chandarana H, Wang H, Tijssen RHN, Das IJ. Emerging Role of MRI in Radiation Therapy. J Magn Reson Imaging. 2018;48(6):1468–78. 10.1002/jmri.26271. PubMed PMID: 30194794; PubMed Central PMCID: PMC6986460. PMC6986460 30194794

[2] Henke LE, Contreras JA, Green OL, Cai B, Kim H, Roach MC, et al. Magnetic Resonance Image-Guided Radiotherapy (MRIgRT): A 4.5-Year Clinical Experience. Clin Oncol (R Coll Radiol). 2018;30(11):720–7. 10.1016/j.clon.2018.08.010. PubMed PMID: 30197095; PubMed Central PMCID: PMC6177300. PMC6177300 30197095

[3] Randall JW, Rammohan N, Das IJ, Yadav P. Towards Accurate and Precise Image-Guided Radiotherapy: Clinical Applications of the MR-Linac. J Clin Med. 2022;11(14):4044. 10.3390/jcm11144044. PubMed PMID: 35887808; PubMed Central PMCID: PMC9324978. PMC9324978 35887808

[4] Ahunbay EE, Peng C, Holmes S, Godley A, Lawton C, Li XA. Online adaptive replanning method for prostate radiotherapy. Int J Radiat Oncol Biol Phys. 2010;77(5):1561–72. 10.1016/j.ijrobp.2009.10.013. PubMed PMID: 20362401. 20362401

[5] Wang G, Wang Z, Guo Y, Zhang Y, Qiu J, Hu K, et al. Evaluation of PTV margins with daily iterative online adaptive radiotherapy for postoperative treatment of endometrial and cervical cancer: a prospective single-arm phase 2 study. Radiat Oncol. 2024;19(1):2. 10.1186/s13014-023-02394-2. PubMed PMID: 38178254; PubMed Central PMCID: PMC10768299. PMC10768299 38178254

[6] Stanley D, Harms J, Kole A, Dobelbower M, McCann C, Levine L et al. Daily adaptive vs. non-adaptive external beam radiation therapy with concurrent chemotherapy for locally advanced non-small cell lung cancer (NSCLC): a prospective randomized trial of an individualized approach for toxicity reduction (ARTIA-Lung). Int J Radiat Oncol Biol Phys. 2023;117(2):e41–2. 10.1016/j.ijrobp.2023.06.739

[7] Keall PJ, Brighi C, Glide-Hurst C, Liney G, Liu PZY, Lydiard S, et al. Integrated MRI-guided radiotherapy - opportunities and challenges. Nat Rev Clin Oncol. 2022;19(7):458–70. 10.1038/s41571-022-00631-3. PubMed PMID: 35440773. 35440773

[8] Hall WA, Paulson ES, van der Heide UA, Fuller CD, Raaymakers BW, Lagendijk JJW, et al. The transformation of radiation oncology using real-time magnetic resonance guidance: A review. Eur J Cancer. 2019;122:42–52. 10.1016/j.ejca.2019.07.021. PubMed PMID: 31614288; PubMed Central PMCID: PMC8447225. PMC8447225 31614288

[9] Chin S, Eccles CL, McWilliam A, Chuter R, Walker E, Whitehurst P, et al. Magnetic resonance-guided radiation therapy: A review. J Med Imaging Radiat Oncol. 2020;64(1):163–77. 10.1111/1754-9485.12968. PubMed PMID: 31646742. 31646742

[10] Møller DS, Holt MI, Alber M, Tvilum M, Khalil AA, Knap MM, et al. Adaptive radiotherapy for advanced lung cancer ensures target coverage and decreases lung dose. Radiother Oncol. 2016;121(1):32–8. 10.1016/j.radonc.2016.08.019. PubMed PMID: 27647459. 27647459

[11] Kishan AU, Ma TM, Lamb JM, Casado M, Wilhalme H, Low DA, et al. Magnetic Resonance Imaging-Guided vs Computed Tomography-Guided Stereotactic Body Radiotherapy for Prostate Cancer: The MIRAGE Randomized Clinical Trial. JAMA Oncol. 2023;9(3):365–73. 10.1001/jamaoncol.2022.6558. PubMed PMID: 36633877; PubMed Central PMCID: PMC9857817. PMC9857817 36633877

[12] Hehakaya C, Van der Voort JR, Lagendijk JJW, Grobbee DE, Verkooijen HM, Moors EHM. Problems and promises of introducing the magnetic resonance imaging linear accelerator into routine care: the case of prostate cancer. Front Oncol. 2020;10(1741). 10.3389/fonc.2020.01741. PMC7493635 32984058

[13] Hall WA, Paulson E, Li XA, Erickson B, Schultz C, Tree A, et al. Magnetic resonance linear accelerator technology and adaptive radiation therapy: An overview for clinicians. CA Cancer J Clin. 2022;72(1):34–56. PubMed PMID: 34792808; PubMed Central PMCID: PMC8985054. 10.3322/caac.21707 PMC8985054 34792808

[14] Guckenberger M, Andratschke N, Chung C, Fuller D, Tanadini-Lang S, Jaffray DA. The Future of MR-Guided Radiation Therapy. Semin Radiat Oncol. 2024;34(1):135–44. 10.1016/j.semradonc.2023.10.015. PubMed PMID: 38105088. 38105088

[15] Corradini S, Alongi F, Andratschke N, Belka C, Boldrini L, Cellini F, et al. MR-guidance in clinical reality: current treatment challenges and future perspectives. Radiat Oncol. 2019;14(1):92. 10.1186/s13014-019-1308-y. PMC6551911 31167658

[16] Stanley DN, Harms J, Pogue JA, Belliveau JG, Marcrom SR, McDonald AM, et al. A roadmap for implementation of kV-CBCT online adaptive radiation therapy and initial first year experiences. J Appl Clin Med Phys. 2023;24(7):e13961. 10.1002/acm2. 13961 PubMed PMID: 36920871; PubMed Central PMCID: PMC10338842. PMC10338842 36920871

[17] Sibolt P, Andersson LM, Calmels L, Sjöström D, Bjelkengren U, Geertsen P, et al. Clinical implementation of artificial intelligence-driven cone-beam computed tomography-guided online adaptive radiotherapy in the pelvic region. Phys Imaging Radiat Oncol. 2021;17:1–7. PubMed PMID: 33898770; PubMed Central PMCID: PMC8057957. 10.1016/j.phro.2020.12.004 PMC8057957 33898770

[18] Bak ME, Jensen NKG, Nøttrup TJ, Mathiesen HF, Roed H, Sjölin M, et al. Clinical experiences with online adaptive radiotherapy of vulvar carcinoma. Acta Oncol. 2023;62(10):1230–8. 2023.2257377 PubMed PMID: 37713179. 10.1080/0284186X.2023.2257377 37713179

[19] Åström LM, Behrens CP, Calmels L, Sjöström D, Geertsen P, Mouritsen LS, et al. Online adaptive radiotherapy of urinary bladder cancer with full re-optimization to the anatomy of the day: Initial experience and dosimetric benefits. Radiother Oncol. 2022;171:37–42. 10.1016/j.radonc.2022.03.014. PubMed PMID: 35358605. 35358605

[20] Byrne M, Achibald-Heeren B, Hu Y, Teh A, Beserminji R, Cai E, et al. Varian ethos online adaptive radiotherapy for prostate cancer: Early results of contouring accuracy, treatment plan quality, and treatment time. J Appl Clin Med Phys. 2022;23(1). 10.1002/acm2.13479 PMC8803282 34846098

[21] Bostel T, Nicolay NH, Grossmann JG, Mohr A, Delorme S, Echner G, et al. MR-guidance – a clinical study to evaluate a shuttle- based MR-linac connection to provide MR-guided radiotherapy. Radiat Oncol. 2014;9(1):12. 10.1186/1748-717X-9-12. PMC3904210 24401489

[22] Kim JY, Tawk B, Knoll M, Hoegen-Saßmannshausen P, Liermann J, Huber PE, et al. Clinical Workflow of Cone Beam Computer Tomography-Based Daily Online Adaptive Radiotherapy with Offline Magnetic Resonance Guidance: The Modular Adaptive Radiotherapy System (MARS). Cancers. 2024;16(6):6. 10.3390/cancers16061210. PMC10969008 38539544

[23] Bostel T, Pfaffenberger A, Delorme S, Dreher C, Echner G, Haering P, et al. Prospective feasibility analysis of a novel off-line approach for MR-guided radiotherapy. Strahlenther Onkol. 2018;194(5):425–34. 10.1007/s00066-017-1258-y. PubMed PMID: 29349601. 29349601

[24] Wilke L, Christ SM, Dal Bello R, Denney E, Fabiano S, Gabrys HS, et al. Safety in MR-enhanced daily adaptive SBRT radiotherapy using a conventional C-arm linear accelerator: an FMEA approach. Z Med Phys. 2025 Jun 2. PubMed PMID: 40461359. 10.1016/j.zemedi.2025.05.002 PMC12766485 40461359

[25] Uher K, Ehrbar S, Tanadini-Lang S, Dal Bello R. Reduction of patient specific quality assurance through plan complexity metrics for VMAT plans with an open-source TPS script. Z Med Phys. 2024;34(4):555–64. 10.1016/j.zemedi.2023.02.003. PMC11624344 37001999

[26] Dal Bello R, Lapaeva M, La Saint-Esteven G, Wallimann A, Günther P, Konukoglu M. Patient-specific quality assurance strategies for synthetic computed tomography in magnetic resonance-only radiotherapy of the abdomen. Phys Imaging Radiat Oncol. 2023;27:100464. 10.1016/j.phro.2023.100464. PubMed PMID: 37497188; PubMed Central PMCID: PMC10366576. PMC10366576 37497188

[27] Winkel D, Bol GH, Werensteijn-Honingh AM, Kiekebosch IH, van Asselen B, Intven MPW, et al. Evaluation of plan adaptation strategies for stereotactic radiotherapy of lymph node oligometastases using online magnetic resonance image guidance. Phys Imaging Radiation Oncol. 2019;9:58–64. 10.1016/j.phro.2019.02.003. PMC7807584 33458426

[28] Tetar SU, Bruynzeel AME, Oei SS, Senan S, Fraikin T, Slotman BJ, et al. Magnetic Resonance-guided Stereotactic Radiotherapy for Localized Prostate Cancer: Final Results on Patient-reported Outcomes of a Prospective Phase 2 Study. Eur Urol Oncol. 2021;4(4):628–34. 10.1016/j.euo.2020.05.007. PubMed PMID: 32536573. 32536573

[29] Nierer L, Eze C, da Silva Mendes V, Braun J, Thum P, von Bestenbostel R, et al. Dosimetric benefit of MR-guided online adaptive radiotherapy in different tumor entities: liver, lung, abdominal lymph nodes, pancreas and prostate. Radiat Oncol. 2022;17(1):53. 10.1186/s13014-022-02021-6. PubMed PMID: 35279185; PubMed Central PMCID: PMC8917666. PMC8917666 35279185

[30] Joye I, Ricardi U, Scorsetti M, Alongi F, Greto D, Christ SM, et al. Local control in oligometastatic cancer patients following metastasis-directed stereotactic ablative radiotherapy: an interim analysis of ESTRO EORTC E2-RADIatE OligoCare cohort. Radiother Oncol. 2026;111545. 10.1016/j.radonc.2026.111545. PubMed PMID: 42019680. 42019680

[31] De Bruycker A, De Neve W, Daisne JF, Vercauteren T, De Gersem W, Olteanu L, et al. Disease Control and Late Toxicity in Adaptive Dose Painting by Numbers Versus Nonadaptive Radiation Therapy for Head and Neck Cancer: A Randomized Controlled Phase 2 Trial. Int J Radiat Oncol Biol Phys. 2024;120(2):516–27. 10.1016/j.ijrobp.2024.01.012. PubMed PMID: 38387811. 38387811

[32] Güngör G, Serbez İ, Temur B, Gür G, Kayalılar N, Mustafayev TZ, et al. Time analysis of online adaptive magnetic resonance-guided radiation therapy workflow according to anatomical sites. Pract Radiat Oncol. 2021;11(1):e11–21. 10.1016/j.prro.2020.07.003. PubMed PMID: 32739438. 32739438

[33] Votta C, Iacovone S, Turco G, Carrozzo V, Vagni M, Scalia A, et al. Evaluation of clinical parallel workflow in online adaptive MR-guided Radiotherapy: A detailed assessment of treatment session times. Tech Innovations Patient Support Radiation Oncol. 2024;29:100239. 10.1016/j.tipsro.2024.100239. PMC10883837 38405058

[34] Tanaka S, Kadoya N, Ishizawa M, Katsuta Y, Arai K, Takahashi H, et al. Evaluation of Unity 1.5 T MR-linac plan quality in patients with prostate cancer. J Appl Clin Med Phys. 2023;24(12):e14122. 10.1002/acm2.14122. PMC10691646 37559561

[35] Rusu DN, Cunningham JM, Arch JV, Chetty IJ, Parikh PJ, Dolan JL. Impact of intrafraction motion in pancreatic cancer treatments with MR-guided adaptive radiation therapy. Front Oncol. 2023;13. 10.3389/fonc.2023.1298099. PMC10756668 38162503

[36] Chick J, Alexander S, Herbert T, Huddart R, Ingle M, Mitchell A, et al. Evaluation of non-vendor magnetic resonance imaging sequences for use in bladder cancer magnetic resonance image guided radiotherapy. Phys Imaging Radiation Oncol. 2023;27:100481. 10.1016/j.phro.2023.100481. PMC10465927 37655122

