# Supplementary material for: MRI-enhanced online adaptive radiotherapy by virtual integration of a standalone MRI simulator and CBCT-based treatment delivery
Source: Radiat Oncol. 2026 Jul 6;21:129. doi: 10.1186/s13014-026-02887-w (PMC13621725; doi:10.1186/s13014-026-02887-w)
Supplement: Supplementary file 1 [file 13014_2026_2887_MOESM1_ESM.docx]

**Supplementary Material**

| **Sequence** | **Duration (min)** | **TR**  **(ms)** | **TE**  **(ms)** | **FA**  **(°)** | **Resolution**  **(mm)** | **FOV**  **(mm^3^)** |
| --- | --- | --- | --- | --- | --- | --- |
| **Localizer** | 0:31 | 13 | 7.3 | 40 | 2.0x2.0x10.0 | 500.0x500.0x640.0 |
| **Large FOV Localizer** | 0:46 | 4.36 | 2.18 | 60 | 2.0x2.0x2.4 | 500.0x453.0x499.2 |
| **T1-DIXON** | 2:08 | 6.23 | 2.39, 4.77 | 15 | 1.6x1.6x2.0 | 500.0x375.0x200.0 |
| **T2-TSE** | 2:54 | 5080 | 94 |  | 0.3x0.3x3.0 | 200.0x200.0x300.0 |

**Table T1:** MRI acquisition protocol used for the Virtual MR-Linac workflow. Abbreviations: TR, repetition time; TE, echo time; FA, flip angle; FOV, field of view; TSE, turbo spin echo.

**Figure F1:** Subgroup dosimetric analysis for the 2-fraction scheme (10 Gy in 2 fractions, n=16). (a–c) Comparison of average target coverage (GTV, PTV) and average dose to 1 cc of the bowel for the base plan, the base plan recalculated on the daily anatomy, and the adapted plan. In this cohort, plan adaptation was associated with a mean increase of 18% in target coverage compared to the recalculated plans

**Figure F2:** Subgroup dosimetric analysis for the 5-fraction scheme (35 Gy in 5 fractions, n=11). (a–c) Comparison of average target coverage (GTV, PTV) and average dose to 1 cc of the bowel for the base plan, the base plan recalculated on the daily anatomy, and the adapted plan. In this cohort, plan adaptation was associated with improved bowel sparing, mitigating increases in D1cc by an average of 16%.

**Figure F3:** Schematic comparison of the MR-Linac workflow (top) and the Virtual MR-Linac workflow (bottom). In the MR-Linac workflow, magnetic resonance imaging (green), treatment plan adaption (purple), and radiotherapy delivery (blue) are performed sequentially on the same machine for each patient. In the Virtual MR-Linac workflow, a diagnostic MR scanner and a conventional linear accelerator are used in parallel, allowing different patients to undergo imaging and treatment at the same time.

**Figure F4:** Example of daily image matching between synthetic CT (sCT) and cone beam CT (CBCT), showing the Gross Tumor Volume (GTV) contoured in blue.

**Figure F5:** Adaptive workflow using standalone MR imaging and a C-arm Linac. Green: steps currently being automated. Yellow: manual steps targeted for future automation. Blue: non-automated steps.
